# Nourishing change in Houston, Texas: exploring grocery shopping behaviours and fruit and vegetable consumption among low-income families in the Brighter Bites program

**DOI:** 10.1017/S136898002400260X

**Published:** 2025-01-09

**Authors:** Victoria Kwentua, Allison Marshall, Ru-Jye Chuang, Jessica Chen, Christine Markham, Mallika Mathur, Mike Pomeroy, Megan Hall, Shreela Sharma

**Affiliations:** 1 Department of Health Promotion and Behavioral Sciences, School of Public Health, The University of Texas Health Science Center at Houston (UTHealth), Houston, TX 77030, USA; 2 Department of Human Science, Georgetown University School of Health, Washington, DC 20007, USA; 3 Department of Epidemiology, Human Genetics, and Environmental Science, The University of Texas Health Science Center at Houston (UTHealth), Houston, TX 77030, USA; 4 Brighter Bites, Houston, TX, USA; 5 The Michael & Susan Dell Center for Healthy Living, The University of Texas Health Science Center at Houston (UTHealth) School of Public Health, 1200 Pressler Street, Houston, TX 77030, USA

**Keywords:** Grocery shopping behaviours, Dietary behaviour change, Nutrition programs, School nutrition interventions, Fruit vegetable consumption

## Abstract

**Objective:**

We qualitatively examine the grocery shopping behaviours and fruit and vegetable consumption of low-income families participating in the Brighter Bites program in Houston, Texas.

**Design:**

We used a single-group observational study design. We used (1) purposive sampling of schools and (2) convenience sampling of parents/caregivers to recruit participants. Research staff conducted three face-to-face qualitative focus groups in Spanish and English. Transcripts were coded using deductive and inductive reasoning.

**Setting:**

Three elementary schools serving low-income families in Houston, Texas, in February-May of 2022.

**Participants:**

Brighter Bites parents/caregivers from the 2021–2022 school year.

**Results:**

Three primary themes emerged: (1) child involvement in grocery shopping – most parents/caregivers shop with their children. Children sometimes bring their own grocery lists, select their produce or help by counting produce; (2) the importance of balancing quality and affordability of fruits and vegetables purchased – both when selecting stores and choosing produce; (3) exposure to new varieties and higher quality of fruits and vegetables through Brighter Bites programming – parents/caregivers reported purchasing new fruits and vegetables as a result of participating in Brighter Bites.

**Conclusion:**

Findings can inform nutrition education programming and policies targeting fruit and vegetable consumption for low-income families. Child involvement may be a good target for nutrition-based behaviour change programs. Nutrition programs and policies should consider both produce affordability and quality. Exposure and opportunities to try new fruits and vegetables can lead to future purchases of new produce. Findings can also inform grocery stores’ efforts to understand low-income families’ purchasing habits, preferences and priorities.

Fruit and vegetable consumption is critical for proper growth, functioning and psychosocial development in children and adolescents^(1,2)^. There is some evidence that vegetable consumption may reduce the risk of weight gain or may be associated with weight loss in an international systematic review that includes studies from the United States (U.S.)^(3,4)^. Beyond weight, fruit and vegetable consumption is associated in the USA with lower mortality, including mortality associated with cancer, CVD and respiratory disease^(3)^. Commonly identified influences on fruit and vegetable consumption include preference, accessibility and availability of fruits and vegetables^(5,6)^. For many families, most energetic and nutrient intake is composed of foods eaten at home; whether cooked from scratch or ready to eat, foods at home are shaped by what foods they have available in their homes that they have purchased or otherwise acquired^(7,8)^. Because of the importance of foods eaten at home, grocery shopping is a critical target for improving dietary behaviours for children and families^(7,8)^.

For low-income families with limited financial resources, grocery shopping is complex. Parents/caregivers seek to provide healthy foods but also need to stretch limited financial resources to provide sufficient food for their households while saving money and reducing waste^(9,10)^. Price, produce quality, store environment and accessibility – such as lack of reliable transportation – are key factors influencing shopping habits in low-income households^(9,11)^. Among a sample of low-income mothers from Twin Cities, Minnesota, U.S., focus group participants expressed awareness of the importance of vegetables at meals and shared perceptions that fresh produce is expensive^(9)^. Further, low-income mothers discussed the importance of purchasing foods they knew their children would eat and that children could make lists and contribute to the shopping efforts in a helpful way^(9)^. Participants from this same sample of low-income mothers also indicated that child involvement in grocery shopping led to higher grocery bills^(9)^. Engaging parents/caregivers, families and schools in multi-component, theoretically based nutrition interventions can enhance home food environments and dietary behaviours for both families and individuals^(12)^. These interventions integrate strategies such as nutrition education with skill building or observational learning experiences.

Brighter Bites is one such intervention that combines multiple strategies to create sustainable, long-term behaviour changes, including teaching families how to shop for and prepare fruits and vegetables. While Brighter Bites has well-documented evidence of influencing dietary behaviours during the temporary provision of produce, it also shows sustained consumption of fruits and vegetables among children afterward. This study elucidates how at-home grocery shopping behaviours may change due to Brighter Bites and will explore whether these behaviours extend to grocery shopping practices, further facilitating improved family dietary behaviours.

A better understanding of Brighter Bites’ impact on grocery shopping is needed to inform programming for sustainable dietary behaviour change and lifestyle habits for children and families. The current qualitative study explores typical or habitual grocery shopping behaviours and factors influencing the shopping behaviours of families participating in the Brighter Bites program in the 2021–2022 school year. The objective of this study was to examine (1) What drives grocery shopping choices among parents/caregivers from a low-income elementary school? and (2) How has Brighter Bites influenced parents/caregivers’ grocery shopping habits? Findings will add insight into in-home parental food environment behaviors such as grocery shopping to identify targets for dietary health promotion programming and contribute to the literature supporting quality improvement for dietary health promotion programs.

## Methods

### Brighter Bites programming

Brighter Bites is an evidence-based, coordinated school health program in the USA that combines access to fresh produce and nutrition education in primarily elementary school settings to improve dietary behaviours among children and parents/caregivers^(13,14)^. Serving predominantly low-income families, the program had school-level eligibility criteria of > 75 % of children enrolled in the free/reduced lunch program. The Brighter Bites program has significantly improved fruit and vegetable intake among participating families and parent/caregiver rules for mealtimes, screen time and meal preparation in the home food environment^(14,15)^. These changes have been maintained over time^(14,15)^. For instance, in a quasi-experimental study involving parent–child dyads, children showed significant improvement in fruit and vegetable intake (*P* = 0·046; *P* = 0·049), while parents/caregivers notably increased their fruit consumption (*P* = 0·032)^(14)^. Additionally, in a program evaluation employing a one-group, pre-post design, home food environment practices were found to persist even after two years of program engagement (*P* < 0·05)^(15)^. Further, Brighter Bites has reduced food insecurity among participants nationwide^(16)^. Improvements in the percentage of program participants reporting food insecurity have been sustained 2 years after program participation in Texas by about 35·4 %, once adjusted for ethnicity^(17)^. Brighter Bites operates in ten U.S. states, reaching over 30 000 families annually across more than 190 schools in the 2022–2023 school year^(18)^.

Brighter Bites is a behavioural theory-based program with a two-pronged approach. The program was designed to improve attitudes and self-efficacy around purchasing, preparing and eating fruits and vegetables and also to build skills for purchasing and preparing healthy recipes using fresh fruits and vegetables^(13)^. Brighter Bites programming consists of three components: (1) 16 bi-weekly produce boxes for children and their families distributed after school (approximately 20 pounds of fresh fruits and vegetables per distribution – about 50 servings), (2) school-based nutrition education delivered by school teachers during the school day for students with posted written/visual materials in the school setting using Coordinated Approach to Child Health^(19,20)^ and (3) nutrition education for parents/caregivers and children including bilingual recipes (Spanish/English), tips on food preparation, food storage, food safety, meal planning and grocery shopping available on the Brighter Bites mobile application and provided in the produce boxes, as well as periodic healthy recipe tastings at participating schools during produce distributions^(13,14)^.

### Study design

We conducted three qualitative focus groups, including current and former participating parents/caregivers from Brighter Bites. We recruited parents from three elementary schools in the greater Houston area in Texas during the 2022 Spring school semester who had participated in Brighter Bites sometime during the 2019–2022 school years. One focus group was conducted at each of the three selected schools. All participants completed printed and verbal consent forms in Spanish or English before participating.

### Participants, recruitment and study setting

We used purposive sampling at the school level, identifying Brighter Bites elementary schools with high parental involvement in the greater Houston area. We used convenience sampling at the individual level, inviting Brighter Bites parents/caregivers who could speak English and/or Spanish to participate in focus groups to gather feedback on families’ grocery shopping habits and how their involvement in Brighter Bites has influenced their habits. Parents/caregivers were recruited at their child’s school through the assistance of a Brighter Bites program staff member. Program staff distributed bilingual flyers to parents/caregivers and shared about the focus groups using word of mouth and through the Brighter Bites texting platform. We recruited a convenience sample of seventeen parents/caregivers from Brighter Bites to participate in the study. Focus groups took place at their child’s Brighter Bites school during the regular school day, typically between 08.00 and 15.30. The timing of the focus groups varied because they were aligned with the Brighter Bites programming schedule at the school, which varies by school depending on space availability and school activities, as well as the overall operational logistics of the Brighter Bites organisation. Two focus groups were held right after drop-off time (08.00–10.00), and one was held right before dismissal time (13.00–14.30). The focus groups were conducted in person using a standardised semi-structured focus group guide in both English (1) and Spanish (2), although all three groups included some bilingual participants.

### Eligibility criteria

To be eligible, participants must have participated in Brighter Bites at one of the three selected schools during the 2019–2022 school years, be the primary grocery shopper of their household, be a parent/caregiver and be at least 18 years or older. Recruitment materials were available in both Spanish and English. Brighter Bites staff and study personnel recruited potential participants.

### Demographics

Demographic data were collected at the beginning of the focus groups using a brief pen-and-paper survey (see Table 2). Demographic data collected included the participant’s self-reported gender, race, age, relationship to the child participating in Brighter Bites, highest level of education completed, employment status and household size. Household food insecurity was assessed using one item: ‘How often in the past three months would you say you were worried or stressed about having enough money to buy nutritious food?’ with response options of never, rarely, sometimes, usually or always^(17)^. Age, grade and gender of the child attending the school and participating in Brighter Bites were also collected. These surveys were administered in both Spanish and English.

**Table 1.** Focus group guide

Topic	Questions
Fruit and vegetable grocery shopping practices	1. Please share how long you have been a participant of Brighter Bites, then tell me how your household typically shops for fruits and vegetables? a. Where do you typically shop? b. Why do you go to this particular store? 2. Tell me about how your kids are involved in your grocery shopping. 3. Think about when you shop for fruits and vegetables – what do you think is the main reason you buy fruits and vegetables? 4. When you go shopping, what are the things that affect the fruits and vegetables that you buy? 5. What keeps you from purchasing fruits and vegetables? [OR What might keep you from buying more fruits and vegetables?] 6. Have you ever shopped online or used a grocery store app for your fruits and vegetables? a. If yes: Where did you shop, and what was your experience with it? b. If no: Why not?
Brighter Bites participation/ Fruit and vegetable shopping activity	7. Let’s think about Brighter Bites programming – receiving weekly produce, tips and recipes to cook the produce. Has participation in the Brighter Bites program affected your fruit and vegetable shopping habits? a. Have you bought more or less fruits and vegetables? Why or why not? b. After you tried it [a fruit or vegetable] with Brighter Bites using our recipe, what was your experience? Did you buy more for your family to eat? c. If these items have not affected your fruit and vegetable shopping habits, why not? d. Think about the month before you joined Brighter Bites. How often did you shop for fruits and vegetables? 8. [Ask if there is time] How often do you shop now for fruits and vegetables? 9. Other than fruits and vegetables, have you changed items you purchase because of something you learned from Brighter Bites? 10. Tell me about any new fruits or vegetables you tried from Brighter Bites that you had never tried before. a. Can you tell me what you might have known about it before trying it with Brighter Bites?
Voucher experience	11. [O*nly asked at schools that received vouchers from* Brighter Bites] Did you receive a voucher/coupon from Brighter Bites to purchase fruits and vegetables at a local grocery store (e.g. Walmart, HEB, Joe-V’s) a. [*If yes*] How did you use the voucher? i. What type of fruits and vegetables did you purchase compared to shopping without a voucher? ii. How much produce did you purchase compared to shopping without a voucher? iii. How often did you shop for fruits and vegetables with a voucher compared to shopping without one?
Final comments	12a. Is there anything else you would like to share about grocery shopping habits? 12b. Is there anything else you would like to share about your experience with Brighter Bites? 12c. Is there anything I should have asked but didn’t?

**Table 2.** Participant demographic characteristics (*n*=17)^*^

Participant demographic variables	Mean	sd
Average Parent/Caregiver Age, mean (sd)^†^	37·1	6·7
	*n*	%
Gender, *n* (%)		
Male	1	5·9
Female	16	94·1
Parent/Caregiver Race/Ethnicity, *n* (%)		
Black or African-American	0	0·0
Mexican-American, Latino, or Hispanic	16	94·1
White, Caucasian, or Anglo	1	5·9
Other^‡^	0	0·0
Preferred home language, *n* (%)		
Most or only English	2	11·8
Both English and Spanish	4	23·5
Most or only Spanish	11	64·7
Other	0	0·0
Relationship to child, *n* (%)		
Mother	15	88·2
Father	1	5·9
Unknown relation	1	5·9
Highest grade completed, *n* (%)		
Never attended school or only attended	1	5·9
Kindergarten	2	11·8
Grades 1–8	1	5·9
Grades 9–11	7	41·2
Grade 12 or GED^§^	3	17·7
College 1–3 years	2	11·8
College 4 years or more (graduate)	1	5·9
Prefer not to answer	0	0·0
	Mean	sd
Household size, mean (sd)		
Total	2.8	1·1
Children under 17 years	2.5	0·9
Elders over 65 years	0	0·0
	*n*	%
Employment status, *n* (%)		
Employed for wages (full or part-time)	1	5·9
Self-employed	1	5·9
Out of work > 1 year	2	11·8
Homemaker/Stay-at-home parent	12	70·6
Prefer not to answer	1	5·9
*‘How often in the past 3 months would you say you were worried or stressed about having enough money to buy nutritious food?’*, *n* (%)		
Never	9	52·94
Rarely	3	17·7
Sometimes	4	23·5
Usually	0	0·0
Always	0	0·0
Prefer not to answer	1	5·9
Child’s gender, *n* (%)		
Male	9	56·3
Female	7	43·8
	Mean	sd
Child’s age, mean (sd)	7·4	2·2
Child’s grade, mean (sd)		
Pre-Kindergarten	3	17·7
Kindergarten	3	17·7
1st grade	2	11·8
2nd grade	5	29·4
3rd grade	1	5·9
4th grade	2	11·8
5th grade	0	0·0
6th grade	0	0·0
7th grade	1	5·9
8th grade	0	0·0

* Sample size (*n*): Some responses had an *n* 16 due to skipped questions or missing data in the survey demographics questions.

† Standard deviation (sd).

‡ Other races/ethnicities included Asian, Native Hawaiian or other Pacific Islander, American Indian or Alaska Native and Mixed Race.

§ General Education Development (GED) certifies that an individual has met skills equivalent to a high school diploma.

### Focus groups

Trained research staff with qualitative research experience conducted in-person focus groups at the three Brighter Bites schools in areas designated by program staff. The focus groups took place in a designated area within the school environment with limited distraction and space for the activity. We verbally presented the written consent document in Spanish and English by participant preference, allowing participants to read it silently for a few minutes. Afterward, parents/caregivers were asked to sign a printed consent form and provide verbal consent for session recording. The focus groups took between 60 and 90 min. Participants were compensated with a $25 Target gift card.

The focus group discussion was conducted using twelve guiding questions with additional probes centred around fruit and vegetable grocery shopping habits and how participation in the Brighter Bites program might have affected the parent’s shopping (Table 1). Topics included past and current grocery shopping practices, barriers and facilitators to fruit and vegetable shopping, online grocery shopping and experiences with Brighter Bites activities, including produce vouchers, nutrition education, healthy recipes and cooking demonstrations. All focus groups were recorded using handheld audio recorders; recordings were transcribed and translated by a professional translation and transcription company. Spanish transcripts were translated into English before coding. Ethical approval was obtained for all research activities.

### Analysis

We used a grounded theory approach and thematic content analysis for the exploratory study. The grounded theory approach guided the development of our focus group questions, which allowed us to generate insights from the data. Thematic content analysis helped us identify and interpret recurring themes within the collected data. Two research staff members trained in qualitative research independently coded the transcripts using inductive and deductive reasoning under the oversight of a PhD-level researcher with qualitative research expertise. Study personnel used deductive reasoning to establish codes based on previous research and an understanding of possible responses to the questions^(21)^. Next, inductive reasoning allowed for any unique codes to emerge that were not expected from the participants. Both trained staff members reviewed the codes to conduct a thematic analysis and identified the themes present in the focus group. Themes with explanatory quotes are presented^(21)^. Dedoose 9.0.62 Qualitative Analysis software was used to manage the focus group data^(22)^.

## Results

### Participant demographic characteristics

Table 2 describes the demographic characteristics of all study participants (n=17). The sample sizes in the three focus groups were n_1_ = 5, n_2_ = 6 and n_3_ = 6. Participants were primarily female (94 %), Hispanic/Latino (94 %) and had at least some high school education (77 %). Most participants reported speaking most or only Spanish (65 %). Most participants were mothers of the child/children participating in Brighter Bites programming (88 %).

The mean household size was 2·8 people. Participants were mainly stay-at-home parents/caregivers (71 %). The duration of Brighter Bites involvement for all participants varied between 0·5 and 2·5 years, and it was assessed as part of the focus group questions when participants introduced themselves. When asked about the persistence of stress or worries concerning purchasing nutritious food in the past 3 months, slightly more than half of participants felt this was never (53 %) an occurrence.

### Focus groups themes

Three major themes were identified: (1) children influence family grocery shopping and participate in family grocery shopping, (2) the importance of finding a balance between quality and affordability of the fruits and vegetables purchased and (3) families were exposed to new and greater quality of fruits and vegetables through Brighter Bites programming. Across focus groups, parents/caregivers understood the importance of fruit and vegetable consumption for their whole family, but especially regarding their children. Parents/caregivers indicated a desire to purchase and provide sufficient fruits and vegetables for their families.

#### Theme 1: Children influenced family grocery shopping and participated in grocery shopping

Parents reported that their children influenced their purchases primarily because parents/caregivers wanted to buy what their children would eat. They also realised the importance of consuming fruits and vegetables for their health. The child’s influence extended beyond preferences, including contributing their own list of fruits and vegetables and helping make the family’s grocery list for the parent. All focus groups mentioned that the child helped pick produce when accompanying the parent/caregiver to the store. Common activities parents/caregivers reported included children counting the produce and assessing the quality by looking at the colour for bruises.

*‘Usually, if they are with me, I let them… pick… some tomatoes.’* - FG1, Participant 1

*‘My girls do participate. They always go. I usually do the shopping in the afternoons when they are there for them to participate in it. And they choose the fruits and vegetables that they like so that they can eat everything and leave nothing. That’s why I take them.’* – FG2, Participant 3

*‘I don’t take my children shopping because I usually do it in the morning, and I only select the fruits that they like. The eldest one, because the little one doesn’t like fruit.’* – FG2, Participant 2

*‘My children … in kinder and pre-kinder, … they make lists as I do. I have my to-do list; I have my shopping list. I have to do it. So, they also have a little notebook or grab a sheet from the printer and make a list of what they want to buy. So, when we go to the store, they go to….saying that they grabbed something, and if they want to eat a snack, then they have to have their fruit. But they already [know] that they have to take their fruits already included in my list or in their list.’* – FG3, Participant 5

#### Theme 2: Balancing the quality and price of the fruits and vegetables purchased was critical

Parents/caregivers reported that quality was the most important factor when buying fruits and vegetables, but they also had to consider price. When money was constrained or they were on a budget, the participants reported changes in their fruit and vegetable purchases and where they shopped. Store choices and food choices depended on both quality and what stores were selling the items at a better price. Parents/caregivers reported selecting seasonal fruits and vegetables for cost reasons and discussed the importance of how long certain fruits or vegetables would last relative to the cost. Parents/caregivers also reported that because Brighter Bites provided some of their produce, they could buy more expensive produce items or purchase a larger quantity of fruits and vegetables other than or in addition to those being provided in the Brighter Bites produce boxes. Though most participants (*n* 14) reported not having experience with Brighter Bites vouchers, the participants (*n* 3) that did receive vouchers reported that the vouchers helped them afford more and better produce by supplementing their budget for grocery purchases^(23)^.

*‘Personally, if it’s out of the price range. That would be another factor after quality.’* – FG3, Participant 3

*‘I’m looking for, as they said, quality, freshness. Because you are not going to buy fruit that is a bit past [overripe], as [participant] says. In my case, it has to last the whole week so it doesn’t go to waste. Like, I grab it, but it’s too ripe or in bad state, and it goes to the trash.’* – FG3, Participant 4

*[Referring to Brighter Bites Voucher]: ‘Fruit and vegetables are more expensive than the snacks … [the voucher] was a good gift because if you didn´t [have it] you are kind of forced to get the $1·99 bag of chips to snack v. a five-dollars blueberries. So, it was good to have a backup like that; you could buy the fresh vegetables regarding the price because you knew it was healthier for the kids. And they liked it, so it was good.’* – FG2, Participant 1

#### Theme 3: Families were exposed to new types and greater overall variety of fruits and vegetables through Brighter Bites programming

The Brighter Bites program helped support parents/caregivers in buying fruits and vegetables they would not typically purchase (e.g. papaya) because it allowed them to try different fruits and vegetables without the financial commitment, so there was less risk. Participants also reported that after trying the new fruits and vegetables (e.g. eggplant), they purchased them again.

*‘I make it [beets from produce bags] in a juice, with carrots and apple. And I drink it, and he [child] drinks it…And he even drinks it by itself. Like, eat it by itself. And I’m like, “What?” I didn’t even know that. He even eats papaya.’* – FG1, Participant 4

*‘I´ve never tried it [eggplant] and I wasn’t going to invest my small budget on something … So, when I got the opportunity in Brighter Bites to try it, now it´s “Okay. We need to get eggplant”. I really enjoyed so it was a good [Inaudible]’* – FG2, Participant 4

*‘Yes, it happened to me with the eggplant. I had never tried it. So, my mother-in-law boiled it, and I think I’m going to buy it again. I think it has made me buy more variety of… fruit or vegetables.’* – FG2, Participant 2

*‘it [participant’s grocery shopping] has changed because there is a bigger variety - there are different things we are trying that we haven’t tried before.’* – FG2, Participant 4

## Discussion

The current study explored the factors influencing grocery shopping choices among parents and caregivers of students from low-income schools, focusing on the potential role of Brighter Bites. Our findings suggest that children greatly influenced their parents’ grocery shopping behaviours. Brighter Bites provides nutrition education to both children and parents, along with distributing produce. This education and tangible provision of fruits and vegetables can encourage families to purchase affordable, high-quality produce even after completing the Brighter Bites program, supporting sustainable dietary behaviour changes.

This study adds nuance and context to existing literature on grocery shopping behaviours among low-income families in high-income countries such as the USA. Additionally, this study adds insight to the literature regarding the impacts of a program designed to change dietary behaviours by providing nutrition education and fresh produce such as Brighter Bites, particularly regarding exposure to new fruits and vegetables and subsequent changes in dietary and purchasing behaviours. The overall understanding of the importance of fruit and vegetable consumption among the parents/caregivers in this study, especially regarding their children’s health, is aligned with existing research^(11)^. Previously, caregivers have identified healthy food purchases, reducing waste and saving money as priorities^(10)^. Among this sample of program participants, there was a high degree of awareness of fruit and vegetable intake recommendations and the benefits of fruits and vegetables, which suggests that low consumption was not a knowledge gap, and that knowledge alone cannot circumvent lack of access or affordability of healthy foods.

Two of the main themes of this study were consistent with existing literature on grocery shopping behaviours – namely, the importance of child involvement as well as the quality and cost of produce. Parents reported that grocery shopping behaviours were influenced by child involvement and personal preferences. Child involvement in grocery shopping was common and important to participants. The findings on child involvement in grocery shopping in this study are closely aligned with research on child involvement regarding children making grocery lists and being helpful at the grocery store, as identified by Wiig et al.^(9)^. While child involvement can present challenges, children could also be a valuable focus for improving grocery shopping behaviours^(10)^.

The importance of produce quality and price identified in this study are also comparable with existing research on low-income families’ grocery shopping behaviours^(11)^. In the current study, personal preferences included prioritising produce quality as a crucial factor, which was impacted by economic circumstances and environmental considerations – the quality, quantity and cost of what was available to participants to purchase in particular stores. Participants described the importance of consuming fruits and vegetables while using their food budgets efficiently – both to provide sufficient fruits and vegetables and to reduce waste.

Notably, Brighter Bites participants reported buying more fruits and vegetables, and seeking produce that they had yet to purchase previously, either due to a supplemented budget or having tried new items through the program, or perhaps a combination. These findings address a gap in the literature on the impacts of programming on grocery shopping behaviours and self-reported dietary behaviouurs. For example, multiple participants specifically reported buying eggplant after trying them in Brighter Bites. This demonstrated that Brighter Bites does increase exposure to and could increase preference for different types of fruits and vegetables, as indicated by reported grocery purchases. Given the importance of exposure and preference for fruits and vegetables as a predictor of fruit and vegetable consumption^(5,6)^, this has important implications in supporting Brighter Bites programming in dietary behaviour change to improve the diet quality of low-income households^(3,4)^.

Overall, parents reported that Brighter Bites positively impacted their grocery shopping behaviours. Part of Brighter Bites programming is nutrition education for parents/caregivers and families. While this qualitative study cannot determine the causation between parental knowledge and fruit and vegetable consumption/purchasing, the study did confirm that parents/caregivers who are part of a produce distribution program understand that eating fruits and vegetables is important for their children. The program can enable parents/caregivers to purchase more fruits and vegetables. Further, Brighter Bites has successfully expanded exposure to new fruits and vegetables. Programs should continue to provide no-cost exposure to new fruits and vegetables to increase the variety of fruits and vegetables consumed, especially among low-income families. Health promotion programming intended to improve the home food environment and grocery shopping behaviours should include children as possible change agents in establishing healthy behaviours.

Additionally, these findings are relevant not only for health promotion and intervention planning but also for grocery store retailers and local growers/farmers. These findings can help retailers understand what new customers are buying and what is influencing their choices. A better understanding of the factors influencing shopping behaviours can inform and positively impact food supply distribution. Retailers should be aware of the importance of increasing access to healthy foods for low-income populations, reaching customers from different cultural backgrounds and connecting growers and vendors with consumers. Ultimately, these findings may also be valuable to those trying to increase fruit and vegetable purchases among low-income families, such as implementers and developers of nutrition assistance programs, policymakers, local growers and grocery retailers. This may represent potential partnership opportunities with aligned priorities to support sustainable health promotion programming. These findings may also inform policy work regarding food pricing and help contextualise the impacts of large-scale economic influences on behaviours that impact health. The findings, however, did not address larger system-level factors influencing where grocery stores are located, nor did they consider issues related to store accessibility, such as transportation challenges, affordability or other environmental factors that affect the availability of fruits and vegetables. Further research and work in these areas is needed.

### Strengths and limitations

This qualitative study is the first to explore the impact of the Brighter Bites program on grocery shopping habits among parents/caregivers from low-income schools. Another strength of this study is that it sought to better understand some individual behaviours (i.e. shopping behaviours and choice), interpersonal (child involvement and family influences) and environmental factors (e.g. store availability, accessibility, produce quality and cost) that could influence dietary behaviours^(2)^. Houston is a large and diverse city with the largest enrollment of schools and families in the Brighter Bites program. While findings from Houston may not be generalisable to all regions, this study still provides critical insight for nutrition programs serving low-income families in other large urban areas.

The relative racial/ethnic homogeneity of the sample may be considered a limitation, as findings may not be generalisable to other groups. The current sample was predominantly Hispanic, so no inferences can be made about racial/ethnic differences in grocery shopping from these data. In particular, no African-American parents/caregivers were in the focus group, although they comprised over 10 % of Brighter Bites participants during this program cycle. According to a study on Brighter Bites parent/caregiver characteristics, most participating African-American parents/caregivers work full-time during the day, making them unable to attend a feedback session during the school day^(24)^. This also occurred in our study, and we consider this another limitation. In other previous research, significant differences have been found in the frequency of grocery shopping at specific types of stores by race/ethnicity and in the links between frequency of shopping and child fruit and vegetable intake^(25)^.

The sample size of 17 was small; however, saturation was reached. We determined saturation was reached when no new themes emerged, which occurred by the third focus group. Recall bias was possible because the parents/caregivers may have inaccurately answered questions about their past grocery shopping routine compared to their current habits during the focus group. Purposive sampling was used at the school level to recruit a convenience sample of parents/caregivers who had children attending a subset of schools in Greater Houston. Most parents/caregivers were also current Brighter Bites volunteers. This may have made them more likely to be engaged with the program and, therefore, more aware of the importance of fruits and vegetables, causing self-selection bias and possibly social desirability bias. These participants may have differed from parents/caregivers who participated less in the programming. Therefore, selection bias should also be considered, since the probability of recruiting parents/caregivers with varying levels of involvement was reduced by the sampling strategy. It should be noted that there could be external factors outside of Brighter Bites programming that were outside the scope of the current study that may also have influenced grocery shopping behaviours. However, the feedback from the sessions will help with formative evaluation and future improvements to nutrition education programs.

### Conclusion

Child influence on and involvement in grocery shopping suggests that child-focused interventions could be a strategic target for interventions aiming to create sustainable behaviour change to support healthy dietary behaviours. Additionally, the demonstrated impact of Brighter Bites on dietary behaviours underscores the importance of further investigation in this area. Future research should consider tailored programming to equip parents/caregivers with strategies and skills to maximise their fruit and vegetable budgets while ensuring access to a wide range of high-quality produce options. The home availability of fruits and vegetables for children and their families^(5,6)^, the proportion of foods eaten from and at home^(7,8)^ and the challenges encountered in recruiting diverse samples for grocery shopping habit research are all significant topics for future studies. Other areas for future research include potential partnerships with grocery retailers and local growers for sustainability and accessibility of fruits and vegetables.
